# Calcium-Activated Chloride Channel ANO1/TMEM16A: Regulation of Expression and Signaling

**DOI:** 10.3389/fphys.2020.590262

**Published:** 2020-11-05

**Authors:** Nickolai O. Dulin

**Affiliations:** Department of Medicine, The University of Chicago, Chicago, IL, United States

**Keywords:** anoctamin-1, TMEM16A, chloride, calcium, signaling

## Abstract

Anoctamin-1 (ANO1), also known as TMEM16A, is the most studied member of anoctamin family of calcium-activated chloride channels with diverse cellular functions. ANO1 controls multiple cell functions including cell proliferation, survival, migration, contraction, secretion, and neuronal excitation. This review summarizes the current knowledge of the cellular mechanisms governing the regulation of ANO1 expression and of ANO1-mediated intracellular signaling. This includes the stimuli and mechanisms controlling ANO1 expression, agonists and processes that activate ANO1, and signal transduction mediated by ANO1. The major conclusion is that this field is poorly understood, remains highly controversial, and requires extensive and rigorous further investigation.

## Introduction

Anoctamin-1 (ANO1), also known as TMEM16A, DOG1 (Discovered On Gastrointestinal stromal tumors 1), ORAOV2 (Oral cancer Overexpressed), and TAOS-2 (Tumor Amplified and Overexpressed) was initially found to be overexpressed in a number of cancer tissues and is thought to contribute to cancer cell growth and tumorigenesis (Bill and Alex Gaither, 2017; Crottes and Jan, 2019). Subsequently, ANO1 was identified as calcium-activated chloride channel (Caputo et al., 2008; Schroeder et al., 2008; Yang et al., 2008). Mutagenesis and structural studies revealed that the ANO1 channel pore is formed by transmembrane helices 3-8; and the residues responsible for Ca^2+^ binding, channel gating and ion selectivity have been identified, providing mechanistic insights on ANO1 activation (Tien et al., 2014; Peters et al., 2015, 2018; Dang et al., 2017; Paulino et al., 2017). Molecular mechanisms of ANO1 activation and regulation are comprehensively reviewed elsewhere (Pedemonte and Galietta, 2014; Contreras-Vite et al., 2016; Jin et al., 2016; Ji et al., 2019). The most recognized cellular functions of ANO1 include the control of cancer cell proliferation, survival and migration by ANO1 (Bill and Alex Gaither, 2017; Crottes and Jan, 2019), secretory function of ANO1 in the epithelia, such as airways, intestines, salivary glands, renal tubules and sweat glands (Jang and Oh, 2014), induction of electrical pacemaker activity of interstitial cells of Cajal in gastrointestinal smooth muscles (Sanders et al., 2012), control of acute pain sensation, chronic pain and anxiety-related behaviors (Oh and Jung, 2016; Cho et al., 2020), and contribution

to contraction of airway and vascular smooth muscles (Oh and Jung, 2016). While the cellular functions of ANO1 are well established, the signaling mechanisms providing these functions appear highly controversial. This review summarizes the current knowledge of mechanisms governing the regulation of ANO1 expression and ANO1-induced intracellular signaling.

### Regulation of ANO1 Expression (Table 1)

Most notably, ANO1 expression is increased in response to several interleukins including IL-4 in bronchial epithelial cells (Caputo et al., 2008; Caci et al., 2015) and human nasal epithelial cells (Kang et al., 2017), IL-6 in pancreatic acinar cells (Wang et al., 2020), and IL-13 in human bronchial epithelial cells (Lin et al., 2015; Qin et al., 2016), human nasal polyp epithelial cells (Zhang et al., 2015) and human esophageal keratinocytes (Vanoni et al., 2020). Epidermal growth factor (EGF) promotes ANO1 expression in colonic epithelial cancer cells (Mroz and Keely, 2012) and in breast cancer cells (Wang et al., 2019). ANO1 expression was increased by angiotensin II in human umbilical vein endothelial cells (HUVECs) (Ma et al., 2017); however, angiotensin II inhibited ANO1 expression in vascular smooth muscle cells (Zhang et al., 2015), demonstrating cell-specific effects. Among the pathological stimuli, ANO1 expression was transiently induced by the bacterial toxin lipopolysaccharide (LPS) in human lung adenocarcinoma A549 cells (Zhang et al., 2014), in cultured mouse lung cancer cell line LA795, and in mouse alveolar epithelial cells *in vivo* after intraperitoneal injection of LPS (Li H. et al., 2016). Bacterial pyocyanin and supernatants from *Pseudomonas aeruginosa* culture stimulated ANO1 expression in human bronchial epithelial cells (Caci et al., 2015).

**TABLE 1:** Regulation of ANO1 expression.

**Stimuli**	**Up/Down (level)**	**Mechanism**	**Cell type**	**Reference**
IL-4	Up (mRNA)		Bronchial epithelial cells	Caputo et al., 2008; Caci et al., 2015
IL-4	Up (protein		Nasal epithelial cells	Kang et al., 2017
IL-4	Up (promoter	STAT6	Colon carcinoma epithelial cells	Mazzone et al., 2015
IL-6	Up (protein	STAT3	Pancreatic acinar cells	Wang et al., 2020
IL-13	Up (mRNA, protein	STAT6	Bronchial epithelial cells	Lin et al., 2015; Qin et al., 2016
IL-13	Up (protein		Nasal polyp epithelial cells	Zhang et al., 2015
IL-13	Up (mRNA, promoter	STAT6	Esophageal keratinocytes	Vanoni et al., 2020
EGF	Up (protein	STAT3	Breast cancer cells	Wang et al., 2019
EGF	Up (mRNA, protein	PKCδ, PI3K	Colonic epithelial cancer cells	Mroz and Keely, 2012
Ang II	Up (protein		Umbilical vein endothelial cells	Ma et al., 2017
Ang II	Down (mRNA, protein	KLF5	Vascular smooth muscle cells	Zhang et al., 2015XH
LPS	Up (mRNA, protein		Lung alveolar epithelial cells	Zhang et al., 2014; Li H. et al., 2016
Pyocyanin	Up (protein		Bronchial epithelial cells	Caci et al., 2015
Serum	Up (protein	STAT6	Gastric cancer cells	Lu et al., 2018
Serum	Up (promoter, protein	Myocardin/SRF	Vascular smooth muscle cells	Zhang et al., 2015XH
Serum	Up (promoter, protein	SP1/MLL1	Gastric cancer cells	Zeng et al., 2019
Serum	Down (promoter, mRNA	Gli1/Gli2	HEK293 cells	Mazzone et al., 2019
5-Aza-CdR	Up (mRNA, protein	DNA di-methylation	Salivary gland cells	Shin et al., 2019
Serum	Down (mRNA, protein	miR-9	Bronchial epithelial cells	Sonneville et al., 2017
Serum	Down (mRNA, protein	miR-9, miR-132 and miR-144	Colorectal cancer cells	Mokutani et al., 2016; Jiang et al., 2019; Park et al., 2019
Serum	Down (mRNA, protein	miR-381	Gastric cancer cells	Cao et al., 2017
Serum	Various isoforms	alternative splicing	Various cell types	Ferrera et al., 2009, 2011; Mazzone et al., 2011; O’Driscoll et al., 2011; Ertongur-Fauth et al., 2014; Ohshiro et al., 2014; Strege et al., 2015; Sung et al., 2016
Serum	Up (cell surface	CLCA1	HEK293	Sala-Rabanal et al., 2015, 2017
Serum	Up (cell surface	14-3-3	HEK293, glioblastoma cells	Lee et al., 2016b
Serum	Up (cell surface	CaMKII	HEK293, glioblastoma cells	Sim et al., 2020
Serum	Down (cell surface	β-COP	HEK293, glioblastoma cells	Lee et al., 2016a

*“Serum” indicates normal cell culture conditions without additional treatments. See the details on cell types in the text.*

Mechanistically, ANO1 expression is regulated at transcriptional, translational and post-translational levels. The signal transducer and activator of transcription (STAT) transcription factors were shown to control ANO1 gene transcription in several studies. STAT3 is important for ANO1 transcription in response to IL-6 in pancreatic acinar cells (Wang et al., 2020) and in response to EGF in breast cancer cells (Wang et al., 2019). EGF-induced ANO1 expression in colonic epithelial cancer cells was also dependent on the activity of protein kinase C-δ (PKCδ) and phosphatidylinositol 3-kinase (PI3K), but the link to STAT3 was not investigated (Mroz and Keely, 2012). ANO1 expression was reported to be dependent on STAT6 in human bronchial epithelial cells (Qin et al., 2016), in esophageal keratinocytes (Vanoni et al., 2020) and in gastric cancer cells (Lu et al., 2018). Others have identified STAT6-binding site on ANO1 promoter and demonstrated its role in IL-4-induced promoter activation by mutagenesis (Mazzone et al., 2015). In vascular smooth muscle cells, ANO1 transcription may be driven by myocardin / serum response factor (SRF) transcription factors (Zhang et al., 2015XH). In gastric cancer cells, the specificity protein 1 (SP1) was shown to bind ANO1 promoter and recruit the mixed lineage leukemia MLL1 protein to promoter region, facilitating histone H3K4 trimethylation, and subsequently promoting ANO1transcription (Zeng et al., 2019). On the other hand, glioma-associated oncogene proteins Gli1/2 were shown to bind and repress ANO1 promoter activation and ANO1 expression in HEK293 model (Mazzone et al., 2019). DNA methylation was also shown to regulate ANO1 expression. An increased expression of ANO1 during salivary gland organogenesis correlated with decreased DNA methylation of ANO1 gene; and demethylating agent 5-aza-2’-deoxycytidine (5-Aza-CdR) drove expression of ANO1 in cultured salivary gland cells (Shin et al., 2019). Conversely, analysis of squamous cell carcinoma [including human papillomavirus (HPV)-positive] samples from two datasets suggested a positive correlation of ANO1 expression with methylation of two CpG islands on ANO1 gene (Finegersh et al., 2017). However, forced hypermethylation of the positively correlated CpG island in normal oral keratinocytes by transfection with HPV oncoprotein E7 that recruits DNA methyl transferases (Sen et al., 2018) had no effect on ANO1 expression, demonstrating a disconnect between the *in vivo* and *in vitro* findings (Finegersh et al., 2017).

ANO1 translation is controlled by a number of microRNAs, including miR-9 in human bronchial epithelial cells (Sonneville et al., 2017), miR-9, miR-132 and miR-144 in colorectal cancer cells (Mokutani et al., 2016; Jiang et al., 2019; Park et al., 2019), and miR-381 in gastric cancer cells (Cao et al., 2017). In addition, alternative splicing of ANO1 in a tissue specific manner affects electrophysiological properties of the channel (Ferrera et al., 2009, 2011; Mazzone et al., 2011; O’Driscoll et al., 2011; Ertongur-Fauth et al., 2014; Ohshiro et al., 2014; Strege et al., 2015; Sung et al., 2016). Post-translationally, for appropriate channel function, ANO1 has to be expressed at the cell surface, which is controlled by other proteins. Secreted calcium-activated chloride channel regulator 1 (CLCA1) stabilizes ANO1 at the cell surface in HEK293 cell model possibly through inhibition of ANO1 internalization (Sala-Rabanal et al., 2015, 2017). Phospho-serine binding protein 14-3-3 directly interacts with ANO1 and also stabilizes ANO1 on cell surface by unknown mechanism, both in heterologous models and in glioblastoma U251 cells (Lee et al., 2016b). Cell surface expression of ANO1 is also promoted by Ca^2+/^Calmodulin-dependent protein kinase II (CaMKII) in heterologous models and in glioblastoma U251 cells (Sim et al., 2020), potentially (but not proven) through phosphorylation of ANO1 by CAMKII (Ayon et al., 2019). In contrast, β-COP, a subunit of Coat Protein Complex I (COPI), directly interacts with ANO1 and reduces its surface expression by promoting the retrograde transport, which was also shown at the endogenous levels of ANO1 and β-COP in U251 cells (Lee et al., 2016a). Importantly, in all these studies the regulated surface expression of ANO1 functionally translated to ANO1 activity.

### Physiological and Pathological Activators of ANO1-Mediated Chloride Currents

Given that ANO1 is a calcium-activated chloride channel, agonists that promote intracellular accumulation of free Ca^2+^ are expected to stimulate ANO1 activity. This was initially demonstrated for G protein-coupled receptor (GPCR) agonists including endothelin-1, angiotensin II, extracellular ATP, histamine and carbachol in human embryonic kidney HEK293 cells co-transfected with cDNAs for ANO1 together with corresponding GPCRs (Yang et al., 2008). Similar finding was made for carbachol in Axolotl oocytes co-injected with *Xenopus* TMEM16A/ANO1 and human M1 muscarinic cRNA (Schroeder et al., 2008). Initial studies also demonstrated ANO1-dependent activation of calcium-dependent chloride currents (CaCC) by extracellular UTP in pancreatic CFPAC-1 cells endogenously expressing ANO1 and purinergic receptors for UTP (Caputo et al., 2008). Subsequently, multiple studies showed the induction of ANO1-mediated CaCC by GPCR agonists in cells with endogenous expression of ANO1 and corresponding GPCRs. Some examples include angiotensin II in cardiac fibroblasts (El Chemaly et al., 2014), basilar artery smooth muscle cells (Li R. S. et al., 2016), and human umbilical vein endothelial cells HUVEC (Ma et al., 2017); endothelin-1 in dorsal root ganglion (DRG) neurons (Cho et al., 2012); epinephrine in Calu-3 human bronchial serous cell model (Banga et al., 2014); carbachol in intestinal epithelial cells (Lee et al., 2019) and in dorsal root ganglion (DRG) neurons (Cho et al., 2012); bradykinin and histamine in DRG neurons (Liu et al., 2010; Cho et al., 2012); and extracellular ATP in FRTL-5 thyroid cells (Iosco et al., 2014). The contribution of ANO1 to CaCC in these studies was determined using pharmacological inhibitors of ANO1 (i.e., T16A_inh_-A01) (Namkung et al., 2011a), siRNA-mediated ANO1 knockdown, or ablation of ANO1 gene. ANO1-mediated CaCC can be also induced by non-GPCR agonists. For example, the receptor tyrosine kinase (RTK) ligand EGF stimulated a transient calcium response and activated CaCC in the pancreatic cancer cell line AsPC-1, which was inhibited by ANO1 knockdown (Crottes et al., 2019). Likewise, brain-derived neurotrophic factor (BDNF), acting through tropomyosin-related kinase (Trk) receptors (Binder and Scharfman, 2004), induced robust Cl^–^ currents, and these effects were blocked by pharmacological inhibition of ANO1 or by ANO1 knockout (Hong et al., 2019). Interestingly, LPS increased currents in whole-cell patch recordings in cultured mouse lung cancer cell line LA795, and this was reduced by ANO1 knockdown; however, the role of calcium in these currents was not demonstrated (Li H. et al., 2016).

ANO1 appears to be a mechano-sensitive channel. As mentioned above, ANO1 is activated by shear stress in biliary cells, likely through a release of extracellular ATP acting through purinergic receptors and an increase in intracellular Ca^2+^ levels (Dutta et al., 2013). In cerebral artery smooth muscle cells, ANO1 is activated by cell swelling and by pressure induced cell stretch through non-selective cation channels and local intracellular Ca^2+^ signaling (Bulley et al., 2012). ANO1 is also a heat sensor, being directly activated by heat over 44 °C; and heat has a synergistic effect on the Ca^2+^ response of ANO1 (Cho et al., 2012). Activation of ANO1 by heat causes depolarization of nociceptive dorsal root ganglion (DRG) neurons, which could be one of the mechanisms for pain sensation (Cho et al., 2012). Importantly, heat induced Cl^–^ currents persisted in DRG neurons isolated from mice deficient for TRPV1 channel—the established heat sensor (Caterina et al., 1997). Finally, ANO1 also senses extracellular proton concentrations. In HEK293 cells transfected with ANO1 cDNA, extracellular acidification enables ANO1 activation in a voltage-independent manner without altering the apparent Ca^2+^ sensitivity of ANO1 (Cruz-Rangel et al., 2017). A number of physiological and pathological processes such as ischemia and cancer progression involve alterations in extracellular pH (Chiche et al., 2010), hence pH sensing of ANO1 could be of high relevance. Furthermore, given that acidification can cause pain in humans (Jones et al., 2004), regulation of ANO1 by protons could be an additional mechanism for pain sensation through ANO1.

### Cell Signaling Mediated by ANO1 (Table 2)

The direction of Cl^–^ currents is determined by membrane potential and the intracellular concentration of Cl^–^ ([Cl^–^]_i_). Most of mature brain neurons contain low [Cl^–^]_i_; and Cl^–^ channel opening would result in Cl^–^ influx and hyperpolarization of the cell, as widely exemplified by the inhibitory action of γ-aminobutyric acid (GABA) that opens Cl^–^ channel GABA_A_ receptors (Fukuda, 2020). In contrast, immature neurons of developing brain contain high [Cl^–^]_i_ resulting in Cl^–^ efflux and cell depolarization upon Cl^–^ channel opening (Fukuda, 2020). Interestingly, in mature cholinergic neurons of medial habenula, ANO1 activity plays excitatory role by contributing to increased frequency of spontaneous firing, presumably due to high [Cl^–^]_i_ in these neurons, although this was not measured in that study (Cho et al., 2020). Noteworthy, activation of the GABA_A_ receptors in these neurons may also increase the firing of action potential (Kim and Chung, 2007). Outside the brain, ANO1 was shown to mediate depolarization and facilitate generation of action potential in spiral ganglion neurons (Zhang et al., 2015XD) and nociceptive DRG neurons (Cho et al., 2012; Deba and Bessac, 2015; Takayama et al., 2015); however, again, the [Cl^–^]_i_ was not measured in these studies.

**TABLE 2:** Cell signaling mediated by ANO1.

**Stimuli**	**ANO1-dependent signaling**	**Cell type**	**Reference**
Spontaneous firing	Depolarization	Cholinergic neurons of medial habenula	Cho et al., 2020
Spontaneous firing	Depolarization	Spiral ganglion neurons	Zhang et al., 2015XD
Heat, Capsaicin, Eact	Depolarization	Nociceptive DRG neurons	Cho et al., 2012; Deba and Bessac, 2015; Takayama et al., 2015
Serum	Depolarization	Interstitial cells of Cajal	Sanders et al., 2012
Serum	Depolarization / Ca^2+^	Smooth muscle cells	Sun et al., 2012; Davis et al., 2013; Dam et al., 2014; Danielsson et al., 2015
Cerulain	Ca^2+^	Pancreatic acinar cells	Wang et al., 2020
Serum	EGFR phosphorylation (Y992); ERK, AKT, CaMKII	Breast cancer cells	Britschgi et al., 2013
EGF	EGFR phosphorylation (Y1016 and Y1092); Ca^2+^; **not PLCγ, AKT, or ERK phosphorylation**	Pancreatic cancer cells	Crottes et al., 2019
EGF	EGFR and STAT3 phosphorylation	Breast cancer cells	Wang et al., 2019
Serum	EGFR protein expression	Head and neck squamous cell carcinoma cells	Bill et al., 2015
Serum	EGFR mRNA and protein expression	Breast cancer cells	Fujimoto et al., 2017
Serum	Ras-GTP binding, phosphorylation of c-Raf, B-Raf, MEK, ERK; **not AKT phosphorylation**	Urinary bladder carcinoma, squamous cell carcinoma cells	Duvvuri et al., 2012
Serum	ERK2 phosphorylation	Hepatoma cells	Deng et al., 2016
IL-13	ERK2 phosphorylation	Bronchial epithelial cells	Qin et al., 2016
Serum	Regulation of p38 and JNK, phosphorylation, **not of ERK phosphorylation**	Prostate cancer cells	Song et al., 2018
Eact	p38 phosphorylation	Lung microvascular endothelial cells	Allawzi et al., 2018
Serum	p38 phosphorylation, **not JNK phosphorylation**	Hepatoma cells	Deng et al., 2016
Serum	AKT phosphorylation	Ovarian cancer cells	Liu et al., 2019
Serum	NFκB activation	Astrocyte cells, glioma cells	Liu et al., 2014
LPS	Inhibition of LPS-induced NFκB	Lung adenocarcinoma cells	Zhang et al., 2014
Eact	mtROS	Lung microvascular endothelial cells	Allawzi et al., 2018
Ang II	NADPH oxidase activity and ROS generation	Umbilical vein endothelial cells	Ma et al., 2017
Ang II	Rho activation, phosphorylation of MLC and MLCP	Smooth muscle cells	Li R. S. et al., 2016

*“Serum” indicates normal cell culture conditions without additional treatments. In bold are the signaling events that are not affected by ANO1 inhibition. See the details on cell types in the text.*

In non-neuronal cells, presumably with high [Cl^–^]_i_ levels, ANO1 promotes depolarization, and facilitates pacemaker activity by interstitial cells of Cajal in intestinal smooth muscle (Sanders et al., 2012), and also contributes to contraction of vascular and airway smooth muscle cells (Sun et al., 2012; Davis et al., 2013; Dam et al., 2014). The latter effect is likely mediated by activation of voltage gated Ca^2+^ channels, as it was inhibited by L-type calcium channel blocker, nifedipine (Sun et al., 2012; Danielsson et al., 2015). Thus, while being activated by Ca^2+^, ANO1 may further promote accumulation of cytosolic free Ca^2+^. There are other mechanisms by which ANO1 activation facilitates an increase in the intracellular Ca^2+^ concentration. ANO1 co-immunoprecipitated with the inositol 1,4,5-trisphosphate receptor (IP3R), was activated by IP3R-mediated Ca^2+^ release, but also contributed to Ca^2+^ release induced by cholecystokinin analog, cerulain, in pancreatic acinar AR42J cells (Wang et al., 2020). ANO1 is also required for EGF-induced store-operated calcium entry (SOCE) in pancreatic cancer cells (Crottes et al., 2019); however, in both latter studies, the precise mechanism by which ANO controls Ca^2+^ signaling was not determined.

Most of the findings on ANO1-dependent cell signaling have emerged from the cancer field; hence, the focus has been on mitogenic/survival signaling. Several reports point to a direct control of EGF receptor (EGFR) activation by ANO1. Knockdown or pharmacological inhibition of ANO1 in ZR75-1 and HCC1954 breast cancer cell lines resulted in a decreased phosphorylation of EGFR at Y992 as well as of phosphorylation of downstream signaling molecules - ERK1/2, AKT and calmodulin kinase II (CaMKII) under normal growth conditions (Britschgi et al., 2013). Knockdown of ANO1 in human pancreatic cancer cells resulted in a significantly inhibited EGF-induced phosphorylation of tyrosine residues Y1016 and Y1092 of EGFR and of Ca^2+^ response to EGF; however, signaling consequences of this are unclear, as EGF-induced phosphorylation of phospholipase Cγ, AKT, and ERK was not affected in that study (Crottes et al., 2019). Knockdown of ANO1 in MCF-7 or T47D breast cancer cells resulted in a reduced tyrosine phosphorylation of EGFR (the site is not reported) and of STAT3 transcription factor in response to EGF, whereas overexpression of ANO1 in T47D cells increased phosphorylation EGFR and STAT3 (Wang et al., 2019). It was reported that ANO1 forms a direct complex with EGFR through the trans/juxtamembrane domain of EGFR in head and neck squamous cell carcinoma (HNSCC) cells; knockdown of ANO1 resulted in greatly reduced EGFR protein levels, which functionally translated to decreased cell proliferation (Bill et al., 2015). Further, knockdown of ANO1 in breast cancer YMB-1 cells also resulted in reduced mRNA levels of HER2 EGFR, suggesting the regulation at a transcriptional level (Fujimoto et al., 2017).

ANO1-induced phosphorylation of ERK1/2 has been demonstrated in urinary bladder carcinoma T24 cells and squamous cell carcinoma SCC-1 cells under normal cell culture growth conditions; and this effect was dependent on ANO1 activity, as the expression of ANO1 K610A mutant (with greatly reduced chloride conductance) had no such effect (Duvvuri et al., 2012). Interestingly, overexpression of ANO1 resulted in activation of upstream signaling molecules of ERK-MAP kinase pathway, including Ras-GTP binding and phosphorylation of c-Raf, B-Raf, and MEK (Duvvuri et al., 2012). In human bronchial epithelial HBE16 cell line, ANO1 expression, elevated either by ANO1 cDNA transfection or by IL-13 treatment, resulted in an increased ERK1/2 phosphorylation; and the effect of IL-13 was abolished by knockdown or pharmacological inhibition of ANO1 (Qin et al., 2016). Knockdown of ANO1 greatly reduced ERK1/2 phosphorylation in human hepatoma cell line SMMC-7721 under standard growth conditions (Deng et al., 2016). However, some studies reported no role of ANO1 in a control of ERK1/2 activation. As such, ANO1 knockdown had no effect on EGF-induced ERK1/2 phosphorylation in human pancreatic cancer cells (Crottes et al., 2019). Silencing ANO1 in prostate cancer PC-3 cells also had no effect on ERK1/2 phosphorylation, but resulted in profound phosphorylation of other MAP kinases, p38, and Jun kinase (JNK), which was associated with increased expression of tumor necrosis factor TNFα and apoptosis (Song et al., 2018). While pharmacologic inhibition of p38 and JNK suggested their role in TNFα induction by ANO1 silencing, TNFα has been known as a powerful inducer of these MAP kinase pathways, which may also explain the effect of ANO1 on p38 and JNK phosphorylation; however, this was not investigated in that study (Song et al., 2018). In contrast, phosphorylation of p38 and apoptosis were induced by pharmacological activator of ANO1, Eact (Namkung et al., 2011b), in rat lung microvascular endothelial cells, which was inhibited by ANO1 knockdown (Allawzi et al., 2018). Similarly, silencing ANO1 in SMMC-7721 hepatoma cell line reduced phosphorylation of p38 (but not JNK) under growth conditions (Deng et al., 2016). The data on ANO1-mediated control of another protein kinase, AKT, relevant to growth and survival, is also controversial. Silencing ANO1 had no effect on AKT phosphorylation induced by EGF in human pancreatic cancer cells (Crottes et al., 2019), or in urinary bladder carcinoma T24 cells and squamous cell carcinoma SCC-1 cells (Duvvuri et al., 2012) under normal cell culture growth conditions. In contrast, ANO1 knockdown resulted in a profound inhibition of AKT phosphorylation in ovarian cancer cell lines SKOV3 and Caov-3 (Liu et al., 2019). Given that ANO1 can mediate CAMKII activation (Britschgi et al., 2013) and that CAMK pathway can activate AKT (Yano et al., 1998), authors postulate that this could be a mechanism for AKT activation by ANO1, but this was not experimentally addressed (Liu et al., 2019). Finally, the data on regulation of the relevant to cell growth and survival nuclear factor-κB (NFκB) transcription factor by ANO1 is also controversial. Overexpression of ANO1 in human astrocyte SVGp12 cells resulted in a decreased phosphorylation of NFκB inhibitor, IκB, accumulation of NFκB in the nucleus and activation of NFκB-dependent gene transcription; whereas knockdown of ANO1 in glioma U87MG cells inhibited NFκB activation (Liu et al., 2014). Similar effect of ANO1 expression on NFκB activation was observed in human bronchial epithelial HBE16 cells (Lin et al., 2015). In contrast, ANO1 overexpression in A549 cells inhibited LPS-induced NF-κB activation and cytokine release (Zhang et al., 2014).

ANO1 appears to control reactive oxygen species (ROS) signaling. Pharmacological activation of ANO1 by Eact in rat lung microvascular endothelial cells resulted in an increased mitochondrial mtROS production (Allawzi et al., 2018), whereas in HUVEC cells, angiotensin II stimulated NADPH oxidase activity and ROS generation, which were significantly reduced by silencing ANO1 (Ma et al., 2017). Mechanistically, ANO1 interacted with Nox2 protein (a key component of NADPH oxidase complex) and reduced the proteasome-dependent degradation of Nox2; however, the role of ANO-mediated chloride conduction was not investigated in this study (Ma et al., 2017). ANO1 was reported to control Rho signaling as related to smooth muscle cell contraction. In basilar artery smooth muscle cells, ANO1 knockdown abolished angiotensin II-induced RhoA activation and phosphorylation of downstream targets, myosin light chain (MLC) and MLC phosphatase, whereas overexpression of ANO1 resulted in opposite effects (Li R. S. et al., 2016). Interestingly, proteomics approach suggested that ANO1 can be in a complex with RhoA, although this was not experimentally confirmed (Perez-Cornejo et al., 2012).

## Discussion

The major message of this review is that the current knowledge of the mechanisms governing the regulation of ANO1 expression and signaling remains poorly understood and is highly controversial. The following major challenges relevant to the topics of this review that could be addressed in future studies are pointed below. (i) The inconstancies between the reports on regulation of ANO1 expression and signaling should be resolved in terms of cell-specificity, laboratory-specific approaches, robustness of the data, and the rigor of the studies. (ii) The proximal consequences of ANO1 activation, such as changes in cell membrane potential and Ca^2+^ signaling are suggested by only a few studies, and this needs to be explored broader. (iii) Activation of ANO1 should logically lead to a change in [Cl^–^]_i_, which has not been (but should be) measured in a majority of studies on ANO1 function. Recently, With-No-Lysine kinase (WNK1) has emerged as a protein whose activity is controlled by Cl^–^ concentration (Piala et al., 2014). WNK1 is expressed in many tissues; hence, ANO1 should control the activity of WNK1 through changes in the concentration of intracellular Cl^–^, which should be investigated, together with the downstream consequences. (iv) The ANO1-mediated signaling should be explored globally through such approaches as transcriptomics, proteomics, metabolomics, etc. (v) Finally, it is crucial to examine how the knowledge of signaling mechanisms obtained in cell culture models translates to pathophysiological processes related to ANO1 function *in vivo*.

## Author Contributions

The author confirms being the sole contributor of this work and has approved it for publication.

## Conflict of Interest

The author declares that the research was conducted in the absence of any commercial or financial relationships that could be construed as a potential conflict of interest. The reviewer OL declared a past co-authorship with the author ND to the handling editor.
